# Temporal Adaptation Enhances Efficient Contrast Gain Control on Natural Images

**DOI:** 10.1371/journal.pcbi.1002889

**Published:** 2013-01-31

**Authors:** Fabian Sinz, Matthias Bethge

**Affiliations:** 1 Department for Neuroethology, University Tübingen, Tübingen, Germany; 2 Werner Reichardt Centre for Integrative Neuroscience, University of Tübingen, Tübingen, Germany; 3 Bernstein Center for Computational Neuroscience, Tübingen, Germany; New York University, United States of America

## Abstract

Divisive normalization in primary visual cortex has been linked to adaptation to natural image statistics in accordance to Barlow's redundancy reduction hypothesis. Using recent advances in natural image modeling, we show that the previously studied static model of divisive normalization is rather inefficient in reducing local contrast correlations, but that a simple temporal contrast adaptation mechanism of the half-saturation constant can substantially increase its efficiency. Our findings reveal the experimentally observed temporal dynamics of divisive normalization to be critical for redundancy reduction.

## Author Summary

The redundancy reduction hypothesis postulates that neural representations adapt to sensory input statistics such that their responses become as statistically independent as possible. Based on this hypothesis, many properties of early visual neurons—like orientation selectivity or divisive normalization—have been linked to natural image statistics. Divisive normalization, in particular, models a widely observed neural response property: The divisive inhibition of a single neuron by a pool of others. This mechanism has been shown to reduce the redundancy among neural responses to typical contrast dependencies in natural images. Here, we show that the standard model of divisive normalization achieves substantially less redundancy reduction than a theoretically optimal mechanism called *radial factorization*. On the other hand, we find that radial factorization is inconsistent with existing neurophysiological observations. As a solution we suggest a new physiologically plausible modification of the standard model which accounts for the dynamics of the visual input by adapting to local contrasts during fixations. In this way the dynamic version of the standard model achieves almost optimal redundancy reduction performance. Our results imply that the dynamics of natural viewing conditions are critical for testing the role of divisive normalization for redundancy reduction.

## Introduction

It is a long-standing hypothesis that the computational goal of the early visual processing stages is to reduce redundancies which are abundantly present in natural sensory signals [1], [2]. Redundancy reduction is a general information theoretic principle that plays an important role for many possible goals of sensory systems like maximizing the amount of information between stimulus and neural response [3], obtaining a probabilistic model of sensory signals [4], or learning a representation of hidden causes [3], [5]. For a population of neurons, redundancy reduction predicts that neuronal responses should be made as statistically independent from each other as possible [2].

**Figure 1:**
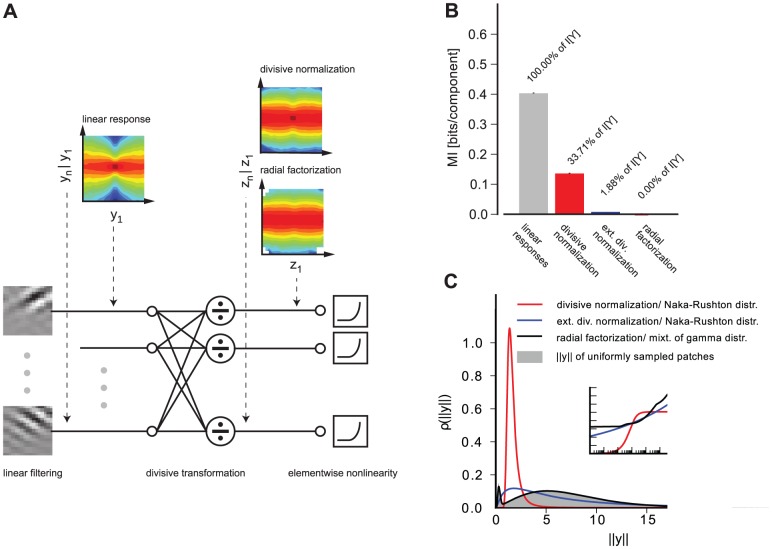
Redundancy reduction and radial distributions for different normalization models. **A**: Divisive normalization model used in this study: Natural image patches are linearly filtered. These responses are nonlinearly transformed by divisive normalization or radial factorization (see text). After linear filtering the width of the conditional distribution 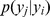 of two filter responses depends on the value of 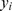 (conditional log-histograms as contour plots). This demonstrates the presence of variance correlations. These dependencies are decreased by divisive normalization and radial factorization. **B**: Redundancy measured by multi-information after divisive normalization, extended divisive normalization, and radial factorization: divisive normalization leaves a substantial amount of residual redundancy (error bars show standard deviation over different datasets). **C**: Distributions on the norm of the filter responses 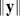 for which divisive normalization (red) and extended divisive normalization (blue) are the optimal redundancy reducing mechanisms. The radial transformation of radial factorization and its corresponding distribution (mixture of five 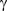-distributions) is shown in black. While radial factorization (inset, black curve) and extended divisive normalization (inset, blue curve) achieve good redundancy reduction, they lead to physiologically implausibly shaped contrast response curves which are mainly determined by their respective radial transformations 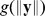 shown in the inset. The radial transformation of divisive normalization is shown for comparison (inset, red curve).

Many prominent neural response properties such as receptive field structure or contrast gain control have been linked to redundancy reduction on natural images [2]. While an appropriate structure of linear receptive fields can always remove all redundancies caused by second order correlations, they have only little effect on the reduction of higher order statistical dependencies [6], [7]. However, one of the most prominent contrast gain control mechanisms—divisive normalization—has been demonstrated to reduce higher order correlations on natural images and sound [8]–[10]. Its central mechanism is a divisive rescaling of a single neuron's activity by that of a pool of other neurons [8, see also Figure 1a].

Recently, *radial factorization* and *radial Gaussianization* have been derived independently by [11] and [12], respectively, based on Barlow's redundancy reduction principle [1]. Both mechanisms share with divisive normalization the two main functional components, linear filtering and rescaling and have been shown to be the unique and optimal redundancy reduction mechanism for this class of transformations under certain symmetry assumptions for the data. Radial factorization is optimal for a more general symmetry class than radial Gaussianization [11], [13] and contains radial Gaussianization as a special case. As a consequence, radial factorization can achieve slightly better redundancy reduction for natural images than radial Gaussianization but the advantage is very small.

Here, we compare the redundancy reduction performance of divisive normalization to that of radial factorization in order to see to what extent divisive normalization can serve the goal of redundancy reduction. Our comparison shows that a non-adapting *static* divisive normalization is not powerful enough to capture the contrast dependencies of natural images. Furthermore, we show that (i) the shape of contrast response curves predicted by radial factorization is not consistent with that found in physiological recordings, and (ii) that for a *static* divisive normalization mechanism this inconsistency is a necessary consequence of strong redundancy reduction. Finally, we demonstrate that a *dynamic* adaptation of the half-saturation constant in divisive normalization may provide a physiologically plausible mechanism that can achieve close to optimal performance. Our proposed adaptation mechanism works via horizontal shifts of the contrast response curve along the log-contrast axis. Such shifts have been observed in experiments in response to a change of the ambient contrast level [14].

## Results

### Measures, Models, Mechanisms

We now briefly introduce divisive normalization, radial factorization, and the information theoretic measure of redundancy used in this study.

#### Redundancy reduction and multi-information

We consider a population of sensory neurons that transforms natural image patches 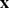 into a set of neural activities 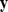 or 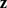. We always use 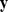 to denote responses to linear filters, and 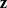 for the output of divisive normalization or radial factorization. The goal of redundancy reduction is to remove statistical dependencies between the single coefficients of 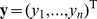 or 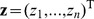.

Redundancy is quantified by the information theoretic measure called *multi-information*

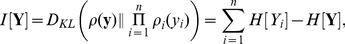
(1)which measures how much the representation differs from having independent components. More precisely, the multi-information is the Kullback-Leibler divergence between the joint distribution and the product of its marginals or, equivalently, the difference between the sum of the marginal entropies and the joint entropy. In case of 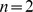 this equals the better known mutual information. If the different entries of 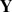 are independent, then its joint distribution equals the product of the single marginals or–-equivalently–-the joint entropy equals the sum of the marginal entropies. Thus, the multi-information is zero if and only if the different dimensions of the random vector 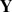 are independent, and positive otherwise. In summary, the multi-information measures all kinds of statistical dependencies among the single coefficients of a random vector. In the Methods Section, we describe how we estimate the multi-information for the various signals considered here.

#### Divisive normalization

From all existing divisive normalization models considered previously in the literature, ours is most closely related to the one used by Schwartz and Simoncelli [9]. It consists of two main components: a linear filtering step and a rescaling step based on the Euclidean norm of the filter responses
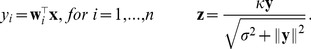
(2)While the linear filters 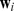 capture the receptive field properties, the rescaling step captures the nonlinear interactions between the single neurons. Most divisive normalization models use filters 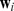 that resemble the receptive fields of complex cells [9], [15], [16]. Therefore, we use filters obtained from training an *Independent Subspace Analysis (ISA)* on a large collection of randomly sampled image patches [15], [16, see also Methods]. ISA can be seen as a redundancy reduction transform whose outputs are computed by the complex cell energy model [17], [18]. For this study, the algorithm has the advantage that it not only yields complex cell-like filter shapes, but also ensures that single filter responses 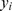 are decorrelated and already optimized for statistical independence. This ensures that the redundancies removed by divisive normalization and radial factorization are the ones that cannot be removed by the choice of linear filters [7], [19].

Several divisive normalization models exist in the literature. They differ, for instance, by whether a unit 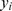 is contained in its own normalization pool, or in the exact form of the rescaling function 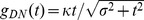 also known as *Naka-Rushton function*. From the viewpoint of redundancy reduction, the former distinction between models is irrelevant because the influence of a single unit on its normalization pool can always be removed by the elementwise invertible transformation 
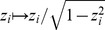
 which does not change the redundancies between the responses [20] (the multi-information is invariant with respect to elementwise invertible transformations). Sometimes, a more general form of the Naka-Rushton function is found in the literature which uses different types of exponents
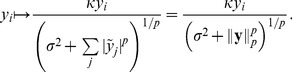
(3)The divisive normalization model considered in this study (equation (2)) differs from this more general version by the type of the norm used for rescaling the single responses: Where equation (3) uses the 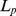-norm 
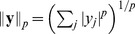
 we use the Euclidean norm. Because radial factorization is defined for the more general 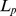-norm (see Methods), all analyses in this paper could be carried out for this more general transform. However, we instead chose to use the Euclidean norm for simplicity and to make our model more comparable to the ones most commonly used in redundancy reduction studies of divisive normalization [9], [20]–[22].

Also note that the Naka-Rushton function is often defined as the 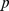
th power of equation (3). However, the form of equation (3) is more common in redundancy reduction studies in order to maintain the sign of 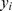. We mention the consequences of this choice in the discussion.

#### Radial factorization

Radial factorization is an optimal radial rescaling for redundancy reduction. We will now briefly introduce radial factorization starting from divisive normalization. For more mathematical details see the Methods Section.

On a population level, the rescaling step of divisive normalization is a nonlinear mapping that changes the Euclidean radius of the filter response population. This can be seen by decomposing divisive normalization into two multiplicative terms
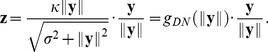
(4)The second term normalizes the response vector 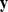 to length one while the Naka-Rushton function in the first term determines the new radius. Since the rescaling 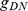 depends only on the norm, the new radius does not depend on any specific direction of 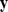.

The redundancy between the coefficients of 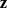 is determined by three factors: The statistics of natural image patches 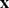 which—together with the choice of filters 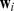
—determine the statistics of 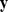, and the radial transformation 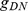. If we allow the radial transformation to be a general invertible transform 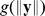 on the Euclidean norm, we can now ask how the different model components can be chosen in order to minimize the redundancy in 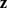.

A substantial part of the redundancies in natural images are second order correlations, which can be removed by linear filters during *whitening* [6]. Whitening does not completely determine the filters since the data can always be rotated afterwards and still stay decorrelated. Higher order decorrelation algorithms like *independent component analysis* use this rotational degree of freedom to decrease higher order dependencies in the filter responses 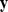 [3]. However, there is no set of filters that could remove all statistical dependencies from natural images [6], [7], because whitened natural images exhibit an approximately spherical but non-Gaussian joint distribution [7], [21], [23], [24]. Since spherical symmetry is invariant under rotation and because the only spherically symmetric factorial distribution is the Gaussian distribution [13], [25], the marginals cannot be independent.

Hence, the remaining dependencies must be removed by nonlinear mechanisms like an appropriate radial transformation 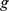. Fortunately, the joint spherically symmetric distribution of the filter responses 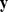 already dictates a unique and optimal way to choose 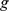: Since a rescaling with 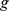 will necessarily result in a spherically symmetric distribution again, 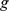 must be chosen such that 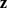 is jointly Gaussian distributed. Therefore, we need to choose 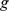 such that 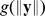 follows the radial distribution of a Gaussian or, in other words, a 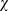-distribution. This is a central point for our study: For a spherically symmetric distribution the univariate distribution on 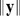 determines higher order dependencies in the multi-variate joint distribution of 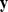. This means that if we restrict ourselves to radial transformations, it is sufficient to look at radial distributions only. The fact that the Gaussian is the only spherically symmetric factorial distribution implies that the coefficients in 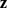 can only be statistically independent if 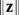 follows radial 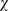-distribution. *Radial factorization* finds a transformation 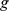 which achieves exactly that by using histogram equalization on the distribution of 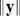 [11], [12, see also Methods]. All these considerations also hold for 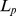-spherically symmetric distributions [11], [13].

Note that this does not imply that the neural responses 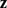 must follow a Gaussian distribution if they are to be independent because the distribution of the single responses 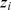 can always be altered by applying an elementwise invertible transformation 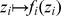 without changing the redundancy. The above considerations only mean that given the two main model components of divisive normalization (and the assumption of spherical symmetry), the best we can do is to choose the 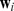 to be whitening filters and 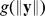 according to radial factorization.

#### Radial factorization and divisive normalization are not equivalent

The goal of this study is to compare the redundancy reduction achieved by divisive normalization and radial factorization. Apart from all similarities between the two models, there is a profound mathematical difference showing that the two mechanisms are not equivalent (as noted by [12]).

Both mechanisms have the form
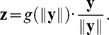
However, the radial rescalings of radial factorization and that of divisive normalization, 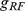 and 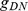, have a different range. Since the 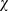-distribution is non-zero on all of 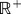 the range of 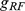 must be 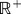 as well. However, in case of divisive normalization, the Naka-Rushton function 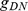 saturates at 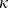. This means that 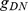 can never transform a radial distribution into a 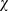-distribution since values beyond 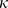 cannot be reached.

While this implies that the two mechanisms are mathematically not equivalent, it could still be that they perform similarly on data if the probability mass of the 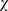-distribution in the range beyond 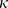 is small. Therefore, we choose 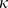 to be the 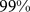 quantile of the 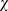-distribution in all our experiments (see Methods).

#### Comparison of the redundancy reduction performance

We compared the amount of redundancy removed by divisive normalization and radial factorization by measuring the multi-information in the plain filter responses 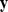 and the normalized responses 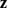 for a large collection of natural image patches (Figure 1b). In both cases the parameters of the radial transformation were chosen to yield the best possible redundancy reduction performance (see Methods). While both divisive normalization and radial factorization remove variance correlations (Figure 1a), the residual amount of dependencies for divisive normalization is still approximately 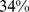 of the total redundancies removed by radial factorization (Figure 1a–b). This demonstrates that divisive normalization is not optimally tailored to the statistics of natural images.

To understand this in more detail, we derived the distribution that 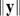 should have if divisive normalization were the optimal redundancy reducing mechanism and compared it to the empirical radial distribution of 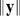 represented by a large collection of uniformly sampled patches from natural images. This optimal distribution for divisive normalization can be derived by transforming a 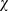-distributed random variable with 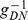 (see Methods). Since 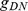 has limited range 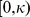 we actually have to use a 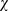-distribution which is truncated at 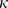. The parametric form of the resulting distribution is given in the Methods Section. We refer to is as *Naka-Rushton distribution* in the following. The parameters of the Naka-Ruston distribution are 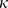 and 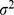. Since 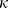 is already determined by fixing the range of 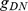 to the 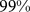 quantile of the 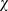-distribution, the remaining free parameter is 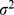. In the Naka-Rushton function 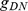 this parameter is called half-saturation constant and controls the horizontal position of the contrast response curve in model neurons.

We fitted 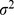 via maximum likelihood (see Methods) and found that even for the best fitting 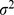 there is a pronounced mismatch between the Naka-Rushton distribution and the empirical distribution given by the histogram (Figure 1c). This explains the insufficient redundancy reduction because the Naka-Rushton distribution expects most of the responses 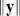 to fall into a much narrower range than responses to natural images do in reality. The Naka-Rushton function 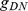 would map the red radial density in Figure 1c perfectly into a truncated 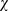-distribution. However, it maps a profound part of the true radial distribution of 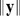 (gray histogram) close to 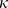, since this part is located to the right of the mode of the Naka-Rushton distribution where it expects almost no probability mass. Additionally, the Naka-Rushton distribution exhibits a small gap of almost zero probability around zero. This gap, however, also contains a portion of empirical distribution. This part gets mapped close to zero. To understand why this leaves significant redundancies, imagine the most extreme case in which all the probability mass of 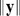 would either be mapped onto 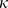 or on onto 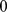. The corresponding distribution on 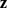 would consist of a point mass at zero and a spherical shell at 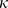. Such a distribution would clearly exhibit strong dependencies.

#### Augmenting divisive normalization by more parameters

It is clear that the suboptimal redundancy reduction performance of divisive normalization is due to its restricted parametric form. Therefore, we explored two options how to increase its degrees of freedom and thereby its redundancy reduction performance: the first option endows static divisive normalization with additional parameters 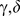, the second option allows for a dynamic temporal adaptation of 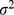.

The simplest way to increase the degrees of freedom in divisive normalization is to introduce two additional parameters in the Naka-Rushton function
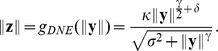
These parameters allow for more flexibility in the scale and shape of the corresponding Naka-Rushton distribution. We label all models that use this parametrization as *extended* in the following. Note that the extended Naka-Rushton function only saturates for 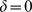. This means that it could in principle transform 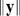 into 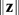 such that 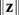 is 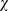-distributed. For 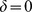 and 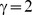, the original Naka-Rushton function is recovered. As before, we derived the corresponding extended Naka-Rushton distribution by transforming a (truncated) 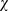-distributed random variable with 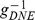. We fitted the resulting distribution to a large collection of 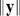, used the maximum likelihood parameters for extended divisive normalization, and measured the redundancy via multi-information in the resulting normalized responses 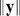.

We found that an extended divisive normalization transform achieves substantially more redundancy reduction and that the extended Naka-Rushton distribution on 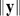 fits the image data significantly better (Figure 1b–c). However, we also find that the best extended Naka-Rushton function for redundancy reduction would yield biologically implausible contrast response curves which capture the firing rate of a neuron upon stimulation with gratings of different contrast at the neuron's preferred spatial frequency and orientation.

In the divisive normalization and the radial factorization model, the shape of the contrast response curve is determined by the shape of the radial rescaling function (Figure 1c, inset) [8]. In contrast to the normal Naka-Rushton function (Figure 1c, inset, red curve), the extended version (Figure 1c, inset, blue curve) exhibits a physiologically unreasonable shape: it starts at a non-zero value, increases without saturation, and does not resemble any sigmoidal shape at all. The non-zero level for low contrasts is a direct consequence of the optimization for redundancy reduction: redundancy reduction implies that the target radial distribution is a (truncated) 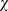-distribution which has only very little probability mass close to zero. Therefore, the radial rescaling function must map the substantial portion of low contrast values in the empirical distribution upwards in order to match the 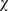-distribution. This results in the immediate non-zero onset. This is a pronounced mismatch to the typical contrast response curves measured in cortical neurons (see Figure 2 in [14]). In fact, the addition of more parameters merely leads to a contrast response curve which is more similar to radial factorization (Figure 1, inset, black) which does not have a plausible shape, too. Therefore, we dismiss the option of adding more parameters to the Naka-Rushton function and turn to the option in which 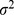 is allowed to dynamically adapt to the ambient contrast level.

**Figure 2:**
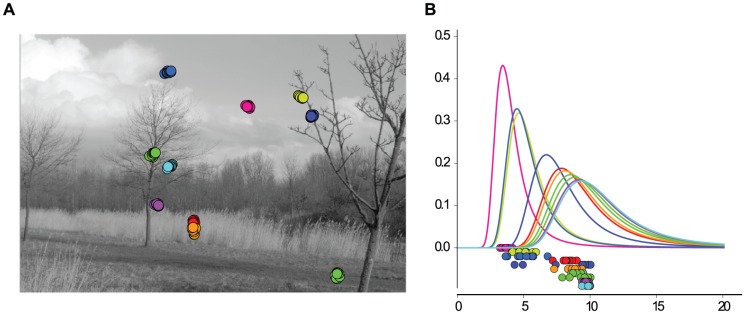
Simulated eye movements and adapted contrast distributions. **A**: Simulated eye movements on a image from the van Hateren database [31]. Local microsaccades are simulated with Brownian motion with a standard deviation of 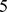
px. In this example, 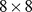 patches are extracted around the fixation location and whitened. **B**: Values of 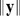 for the extracted patches plotted along the 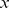-axis. Vertical offset was manually introduced for visibility. Colors match the ones in **A**. The different curves are the maximum likelihood Naka-Rushton distributions estimated from the data points of the same color.

#### Dynamic divisive normalization

Previous studies found that single neurons adapt to the ambient contrast level via horizontal shifts of their contrast response curve along the log-contrast axis [8], [14]. In the divisive normalization model, this shift is realized by changes in the half-saturation constant 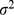. This means, however, that there is not a single static divisive normalization mechanism, but a whole continuum whose elements differ by the value of 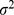 (Figure 2). This is equivalent to a continuum of Naka-Rushton distributions which can be adapted to the ambient contrast level by changing the value of 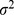. Since this kind of adaptation increases the degrees of freedom, it could also lead to a better redundancy reduction performance.

In order to investigate adaptation to the local contrast in a meaningful way, we used a simple model of saccades and micro-saccades on natural images to sample fixation locations and their corresponding filter responses 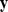 (see Methods). Previous studies on redundancy reduction with divisive normalization [9], [11], [12] ignored both the structure imposed by fixations between saccades in natural viewing conditions, and the adaptation of neural contrast response curves to the ambient contrast level via the adaptation of 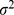 [14]. Figure 2 shows an example of simulated eye movements on a natural image from the van Hateren database. For each sample location, we computed the corresponding values of 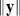 and fitted a Naka-Rushton distribution to it. The right hand side show the resulting Naka-Rushton distributions. One can see that the mode of the distribution shifts with the location of the data, which itself depends on the ambient contrast of the fixation location.

A dynamically adapting 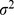 predicts that the distribution of 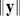 across time should be well fit by a mixture of Naka-Rushton distributions. Let 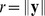 (we use 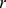 to emphasize that the radial distribution is a univariate density and not a multivariate density on 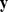), then averaged over all time points 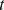, the distribution of 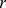 is given by
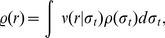
(5)where 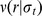 denotes a single Naka-Rushton distribution at a specific point in time.

**Figure 3:**
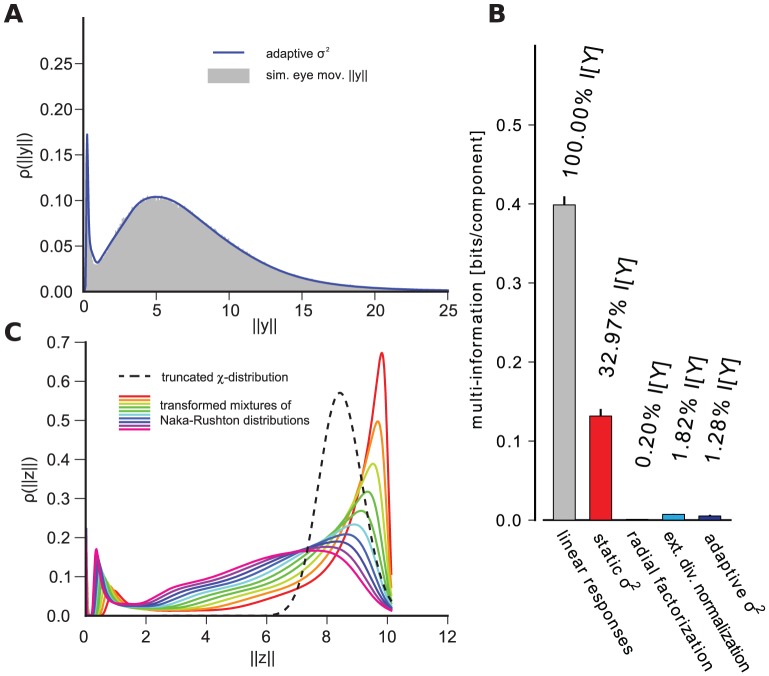
Radial distribution and redundancy reduction achieved by the dynamically adapting model. **A**: Histogram of 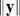 for natural image patches sampled with simulated eye movements: The distribution predicted by the dynamically adapting model closely matches the empirical distribution. **B**: Same as in Fig. 1B but for simulated eye movement data. The dynamically adapting 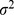 achieves an almost optimal redundancy reduction performance. **C**: Each colored line shows the distribution of a random variable from 3A transformed with a Naka-Rushton function. Different colors correspond to different values of 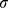. The dashed curve corresponds to a truncated 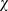-distribution. A mixture of the colored distributions cannot resemble the truncated 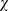-distribution since there will either be peaks on the left or the right of the dashed distribution that cannot be canceled by other mixture components.

We fitted such a mixture distribution to samples 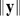 from simulated eye movements (see Methods). Figure 3a shows that the mixture of Naka-Rushton distributions fits the empirical data very well, thus confirming the possibility that a dynamic divisive normalization mechanism may be used to achieve optimal redundancy reduction.

The next step is to find an explicit dynamic adaptation mechanism that can achieve optimal redundancy reduction. To this end, we sought for a way to adapt 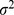 such that the redundancies between the output responses 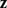 were small. Our temporally adapting mechanism chooses the current 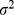 based on the recent stimulation history by using correlations between the contrast values at consecutive time steps. We estimated 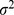 for the present set of filter responses 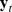 from the immediately preceding responses 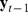 by sampling 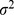 from a 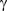-distribution whose parameters were determined by the mean and the variance of the posterior 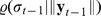 which was derived from the mixture distribution above (see Methods). We found that this temporal adaptation mechanism significantly decreased the amount of residual redundancies to about 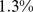 (Figure 3B). Note that the proposed mechanism is a simple heuristic that does not commit to a particular biophysical implementation of the adaptation, but it demonstrates that there is at least one mechanism that can perform well under realistic conditions a neural system would face.

**Figure 4:**
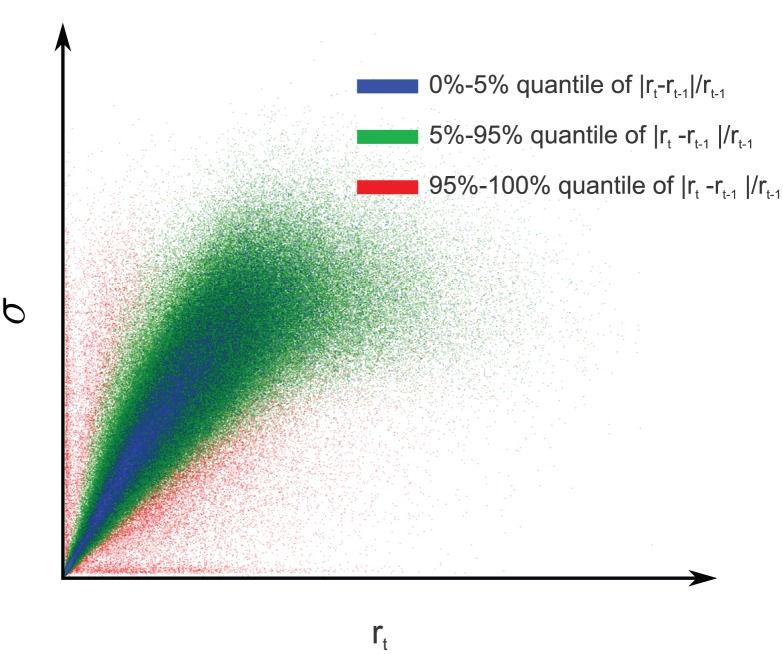
Dynamics of the adaptive 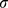. The scatter plot shows the values of 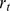 plotted against the 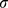 used to transform 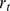 in the dynamic divisive normalization model. The two values are clearly correlated. This indicates that the shift of the contrast response curve, which is controlled by 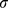, tracks the ambient contrast level, which is proportional to 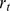. Single elements in the plot are colored according to the quantile the value of 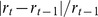 falls in. When the ambient contrast level changes abruptly (e.g. when a saccade is made), this value is large. If the ambient contrast level is relatively stable (e.g. during fixation), this value is small. In those situations (blue dots), 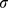 and 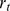 exhibit the strongest proportionality.

Looking at the joint dynamics of 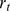 and its 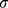 (Figure 4) we find them to be strongly and positively correlated. Therefore, a higher value of 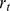 is accompanied by a higher value of 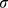. This is analogous to the adaptation of neural contrast response curves observed in vivo where a higher contrast (higher 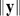) shifts the contrast response curve to the right (higher 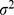), and vice versa [14].

In order to demonstrate that improved redundancy reduction is a true adaptation mechanism which relies on correlations between temporally subsequent sample, we need to preclude the possibility that 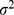 can be sampled independently (i.e. context independent). For strong redundancy reduction, the normalized responses 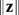 should follow a (possibly truncated) 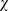-distribution (see Methods). The history-independent choice of 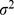 predicts that this truncated 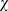-distribution should be expressible as a mixture of distributions that result from transforming random variables, that follow a mixture of Naka-Rushton distributions from Figure 3C, with Naka-Rushton functions for different values of 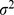 (see Methods for the derivation). We transformed the input distribution with Naka-Rushton functions that differed in the value of 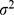 (Figure 3C, colored lines). Different colors in Figure 3C refer to different values of 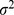. If 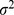 was history-independent, a positively weighted average of the colored distributions should be able to yield a truncated 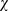-distribution (Figure 3C, dashed line). It is obvious that this is not possible. Every component will either add a tail to the left of the 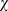-distribution or a peak to the right of it. Since distributions can only be added with non-negative weight in a mixture, there is no way that one distribution can make up for a tail or peak introduced by another. Therefore, 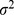 cannot be chosen independently of the preceding stimulation, but critically relies on exploiting the temporal correlation structure in the input.

## Discussion

In this study we have demonstrated that a *static* divisive normalization mechanism is not powerful enough to capture the contrast dependencies of natural images leading to a suboptimal redundancy reduction performance. Static divisive normalization could only exhibit close to optimal performance if the contrast distribution of the input data would be similar to a Naka-Rushton distribution that we derived in this paper. For the best fitting Naka-Rushton distribution, however, the interval containing most of the probability mass is too narrow and too close to zero compared to the contrast distribution empirically found for natural image patches. A divisive normalization mechanism that uses the 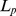-norm as in equation (3) instead of the Euclidean norm would suffer from the same problem because the Naka-Rushton distribution for 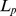-norms other than 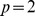 would have similar properties. However, the good performance of extended divisive normalization demonstrates that it is not necessary to model the contrast distribution perfectly everywhere but that it would be sufficient to match the range where most natural contrasts appear (Figure 1C).

Not every mapping on natural contrasts that achieves strong redundancy reduction is also physiologically plausible: We showed that the extended static mechanism yields physiologically implausible contrast response curves. Extending the static mechanism of divisive normalization for better redundancy reduction simply makes it more similar to the optimal mechanism and, therefore, yields implausible tuning curves as well. We thus suggested to consider temporal properties of divisive normalization and devised a model that can resolve this conflict by temporally adapting the half-saturation constant 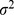 using temporal correlations between consecutive data points caused by fixations.

Another point concerning physiological plausibility is the relationship between divisive normalization models used to explain neurophysiological observations, and those used in redundancy reduction studies like ours. One very common neurophysiological model was introduced by Heeger [8] which uses half-squared instead of linear single responses:
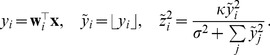
(6)In order to represent each possible image patch this model would need two neurons per filter: one for the positive part and one for the negative part 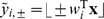. Of course, these two units would be strongly anti-correlated since only one can be nonzero at a given point in time. Therefore, taking a redundancy reduction view requires considering the positive and the negative part. For this reason it is reasonable to use 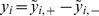 as the most basic unit and define the normalization as in equation (2). Since 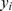 and 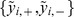 are just two different representations of the same information, the multi-information between 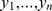 is the same as the multi-information between different tuples 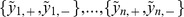. Apart from this change of viewpoint, the two models are equivalent, because the normalized half-squared response of equation (6) can be obtained by half-squaring the normalized response of equation (2). Therefore, a model equivalent to the one in equation (6) can be obtained by using the model of equation (2) and representing its responses 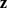 by twice as many half-squared coefficients afterwards.

Previous work on the role of contrast gain control for efficient coding has either focused on the temporal domain [26], [27], or on its role in the spatial domain as a redundancy reduction mechanism for contrast correlations in natural images [9], [11], [12]. Our results emphasize the importance of combining both approaches by showing that the temporal properties of the contrast gain control mechanism can have a critical effect on the redundancies that originate from the spatial contrast correlations in natural images. Our analysis does not commit to a certain physiological implementation or biophysical constraints, but it demonstrates that the statistics of natural images require more degrees of freedom for redundancy reduction in a population response than a classical static divisive normalization model can offer. Our heuristic mechanism demonstrates that strong redundancy reduction is possible with an adaptation mechanism that faces realistic conditions, i.e. has only access to stimuli encountered in the past.

As we showed above, biologically plausible shapes of the contrast response curve and strong redundancy reduction cannot be easily brought together in a single model. Our dynamical model offers a possible solution to this problem. To what extent this model reflects the physiological reality, however, still needs to be tested experimentally.

The first aspect to test is whether the adaptation of the half-saturation constant reflects the temporal structure imprinted by saccades and fixations as predicted by our study. Previous work has measured adaptation timescales for 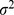 [14], [28]. However, these measurements are carried out in anesthetized animals and cannot account for eye movements. Since our adaptation mechanism mainly uses the fact that contrasts at a particular fixation location are very similar it predicts that that adaptive changes of 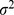 should be seen from one fixation location to another when measured under natural viewing conditions.

**Figure 5:**
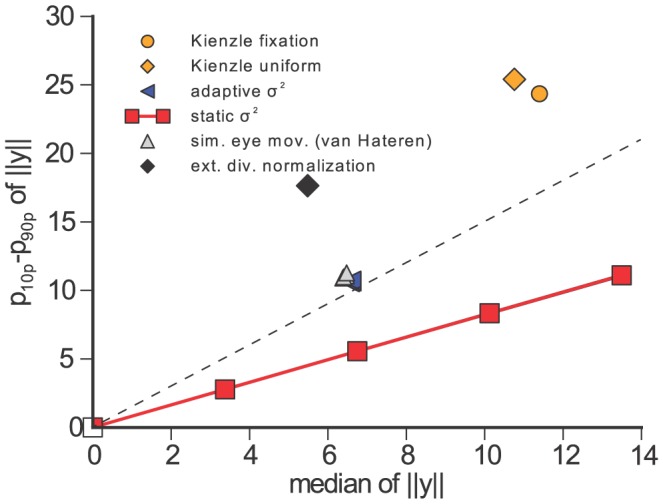
Median vs. width of 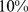 to 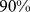 percentile interval of the models shown in Figure 3b. The red line corresponds to a static 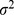 for different values of 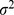, the blue triangles correspond to the temporally adapting 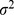, the orange markers correspond to uniformly sampled (diamond) and fixational image patches with Brownian motion micro-saccades (circle) from Kienzle et al. [29], the gray markers to simulated eye movement datasets from van Hateren image data [31], and the black marker to the optimal extended divisive normalization model. All transforms that yield a strong redundancy reduction have models that exhibit a ratio greater than 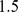 (dashed lines).

The mechanism we proposed is only one possible candidate for a *dynamic* contrast gain control mechanism that can achieve strong redundancy reduction. We conclude the paper with defining a measure that can be used to distinguish contrast gain control mechanisms that are likely to achieve strong redundancy reduction from those that do not. As discussed above, a necessary condition for strong redundancy reduction is that the the location and the width of the distribution of 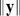 implied by a model must match the distribution of unnormalized responses 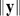 determined by the statistics of natural images. In order to measure the location and the width of the distributions in a way that does not depend on a particular scaling of the data, we plotted the median against the width of the 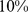
–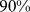
–percentile interval (Figure 5). For the empirical distributions generated by the statistics of the image data we always found a ratio greater than 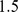. We also included a dataset from real human eye movements by Kienzle et al. to ensure the generality of this finding [29] as real fixations could introduce a change in the statistics due to the fact that real observers tend to look at image regions with higher contrasts [30]. All models that yield strong redundancy reduction also exhibit a ratio greater than 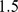. Thus, the ratio of the median to the width of the contrast distribution is a simple signature that can be used to check whether an adaptation mechanism is potentially powerful enough for near-optimal redundancy reduction.

## Methods

The code and the data are available online under http://www.bethgelab.org/code/sinz2012.

### Data

#### van Hateren data

For the static experiments, we used randomly sampled 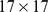 patches from the van Hateren database [31]. For all experiments we used the logarithm of the raw light intensities. We sampled 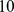 pairs of training and test sets of 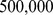 patches which we centered on the pixel mean.

For the simulated eye movements, we also used 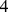 pairs of training and test sets. For the sampling procedure, we repeated the following steps until 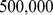 samples were drawn: We first drew an image randomly from the van Hateren database. For each image, we simulated ten saccades to random locations in that image. For each saccade location which was uniformly drawn over the entire image, we determined the number 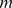 of patches to be sampled from around that location by 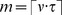 where 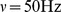 was the assumed sampling frequency and 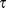 was a sample from an exponential distribution with average fixation time 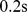 (i.e. 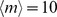). The actual locations of the patches were determined by Brownian motion starting at the saccade location and then propagating with a diffusion constant of 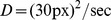. This means that each patch location was drawn relative to the previous one based on an isotropic Gaussian centered at the current location with a standard deviation of 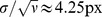.

#### Kienzle data

The van Hateren database is a standard dataset for static natural image statistics. To make sure that our results also hold for real fixations, we sampled data from the images used by Kienzle et al. [29]. We computed the 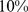 and 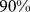 percentiles, as well as the width of the interval between them, for both datasets for Figure 5.

We constructed two datasets: One where the patches were uniformly drawn from the images, and one where we again used Brownian motion with a similar standard deviation around human fixation spots to simulate human fixational data. We applied the same preprocessing as for the van Hateren data: centering and whitening.

### Models

Both the divisive normalization model and the optimal radial factorization consist of two steps: a linear filtering step and a radial rescaling step (Table 1). In the following, we describe the different steps in more detail.

**Table 1:** Model components of the divisive normalization and radial factorization model: Natural image patches are filtered by a set of linear oriented band-pass filters.

	divisive normalization model	radial factorization
filtering	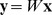	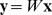
normalization	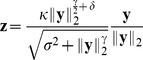	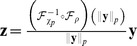
	(static case 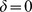 and 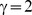 )	

The filter responses are normalized and their norm is rescaled in the normalization step.

#### Filters

The receptive fields of our model neurons, i.e. the linear filters of our models, are given by the rows of a matrix 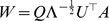. In summary, the filters are obtained by (i) projecting the data onto the 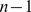 dimensional subspace that is insensitive to the DC component in the image patches, (ii) performing dimensionality reduction and whitening using principal component analysis, and (iii) training an independent subspace analysis algorithm (ISA) to obtain 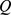:

- The projection of the data onto the 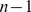 dimensional subspace that is insensitive to the DC component is achieved via the matrix 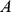. This matrix is a fixed matrix for which the coefficients in each row sum to zero and all rows are mutually orthogonal. The matrix we used has been obtained via a QR-decomposition as described in the Methods Section of [7].
- The dimensionality reduction and whitening is achieved by 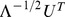. The matrix 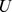 contains the principal components of 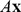 such that 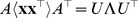. As it is common practice, we kept only the first 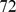 principal components to avoid “noisy” high frequency filters. However, our analysis would also be valid and lead to the same conclusions if we kept the full set of filters.
- The last matrix 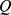 is constrained to be an orthogonal matrix because the covariance of whitened data remains white under orthogonal transformations. This additional degree of freedom is used by Independent Subspace Analysis (see below) to optimize the filter shapes for redundancy reduction beyond removing second-order correlations. While the matrix 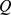 has a large effect on the particular filter shapes, the same results would have been obtained with any type of whitening filter, i.e. for any orthogonal matrix 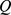, because they only differ by an orthogonal rotation. Since we use the Euclidean norm in the divisive normalization model, the rotation would not change the norm of the filter responses and therefore all radial distributions would be the same. The only aspect in our analysis for which the filter choice would make a (small) difference is the multi-information of the raw filter responses. When using ICA filter, the multi-information could be a bit lower. However, since even for rather drastic changes of filter shapes (within the class of whitening filters) there is only a small effect on redundancy reduction [6], the particular choice of filter shapes does not affect any of our conclusions. The same is true for any choice of parametric filters as long as the covariance matrix of the filter responses is proportional to the identity matrix. Since the second-order correlations provide the dominant contribution to the multi-information any substantial deviation from the class of whitening filters is likely to yield suboptimal results.

The independent subspace analysis (with two-dimensional subspaces) used to obtain the matrix 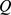 is based on the model by Hyvärinen [16]:
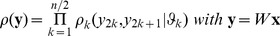
(7)where 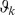 denotes the list of free parameters for each 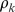. More specifically, 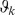 consists of the value 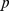 for the 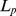-norm and the parameters of the radial distribution for each of the 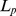-spherically symmetric distributions. Each single 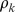 was chosen to be a two-dimensional 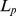-spherically symmetric distribution [32]

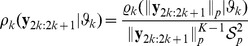


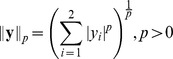
with a radial 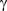-distribution 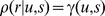 with shape 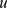 and scale 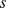. Therefore, the parameters 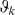 were given by 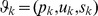. In the denominator, 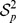 denotes the surface area of the 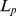-norm unit sphere in two dimensions [32]. During training, we first fixed 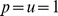; after initial convergence, we retrained the model with free 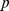 and 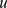.

The likelihood of the data under equation (7) was optimized by alternating between optimizing 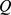 for fixed 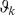, and optimizing the 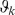 for fixed 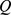. The gradient ascent on the log-likelihood of 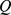 over the orthogonal group used the backprojection method by Manton [19], [33], [34]. Optimizing over 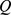 yields filter pairs that resemble quadrature pairs like in the energy model of complex cells [17], [18].

### Radial rescaling

#### Optimal contrast gain control: radial factorization

In the following we describe the general mechanism of radial factorization. The spherical symmetric case mostly used in this study is obtained by setting 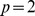.

Radial factorization is the optimal redundancy reduction mechanism for 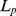-spherically symmetric distributed data [11], [32]. Samples from 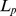-spherically symmetric distributions with identical 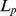-norm 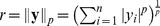 are uniformly distributed on the 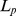-sphere with that radius. A radial distribution 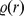 determines how likely it is that a data point is drawn from an 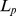-sphere with that specific radius. Since the distribution on the sphere is uniform for any 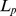-spherically symmetric distribution, the radial distribution 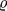 determines the specific type of distribution. For example, 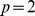 and 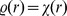 yields an isotropic Gaussian since the Gaussian distribution is spherically symmetric (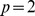) and has a radial 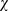-distribution (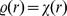). One can show that, for a fixed value of 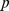, there is only one type of radial distribution such that the joint distribution is factorial [13]. For 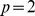 this radial distribution is the 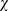-distribution corresponding to a joint Gaussian distribution. For 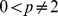, the radial distribution is a generalization of the 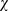-distribution and the joint distribution is the so called 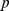-generalized Normal [35].

Radial factorization is a mapping on the 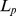-norm 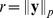 of the data points that transforms a given source 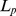-spherically symmetric distribution into a 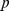-generalized Normal. To this end, it first models the distribution of 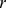 with a flexible distribution 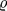 and then nonlinearly rescales 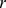 such that the radial distribution becomes a generalized 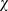-distribution. This is achieved via histogram equalization 
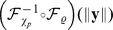
 where the 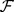 denote the respective cumulative distribution functions. On the level of joint responses 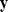, radial factorization first normalizes the radius to one and then rescales the data point with the new radius:
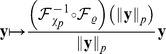
In our case 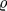 was chosen to be a mixture of five 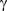-distributions.

When determining the optimal redundancy reduction performance on the population response, we set 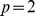 in order to use the same norm as the divisive normalization model. Only when estimating the redundancy of the linear filter responses, we use 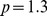 [11].

Note that the divisive normalization model and the radial factorization model used in this study are invariant with respect to the choice of 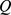 since the Euclidean norm (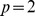) is invariant under orthogonal transforms. However, the choice of 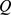 would affect the redundancies in the plain filter responses 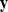 in Figure 1B. But even if we had chosen a different 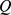, i.e. another set of whitening filters, the redundancy between the coefficients of 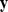 would not vary much as previous studies have demonstrated [6], [7].

#### Divisive normalization model and Naka-Rushton distribution

We use the following divisive normalization transform
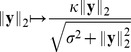
which is the common model for neural contrast gain control [8] and redundancy reduction [9].

Divisive normalization acts on the Euclidean norm of the filter responses 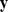. Therefore, divisive normalization can only achieve independence if it outputs a Gaussian random variable. While in radial factorization the target and source distribution were fixed, and the goal was to find a mapping that transforms one into the other, we now fix the mapping to divisive normalization, the target distribution on the normalized response 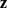 to be Gaussian (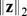 to be 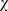-distributed) and search for the corresponding source distribution that would lead to a factorial representation when divisive normalization is applied. Since divisive normalization saturates at 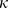, we will actually have to use a truncated 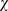-distribution on 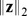. 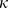 becomes the truncation threshold. Note that radial truncation actually introduces some dependencies, but we keep them small by choosing the truncation threshold 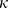 to be the 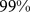 percentile of the radial 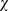-distribution which is approximately 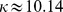. The 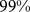 was chosen to keep the target distribution close to a factorial Gaussian. However, it could still be that another cut-off (value of 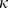) leads to a better redundancy reduction even though the target distribution is less factorial for lower values of 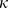 (quantiles lower than 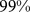). We made sure that this is not the case by choosing different values of 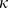, computing the best 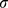 via a maximum likelihood fit of a Naka-Rushton distribution (see below), and estimating the multi-information in the transformed outputs. We found that the choice of 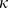 has virtually no effect on the residual multi-infomation (it varies by 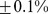 for 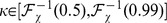 and takes its optimum within this interval). Therefore, we kept the 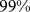 choice as it is most similar to the target distribution of radial factorization.

Note also that choosing a Gaussian target distribution does not contradict the finding that cortical firing rates are found to be exponentially distributed [36] since each single response 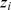 can always be transformed again to be exponentially distributed without changing the redundancy of 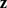.

The distribution on 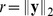 such that
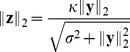
is truncated 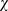-distributed can be derived by a simple change of variables. In the resulting distribution
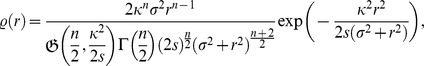
the truncation threshold 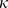, the half-saturation constant 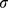, and the scale of the 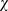-distribution become parameters of the model. The parameter 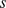 of the Naka-Rushton distribution controls the variance of the corresponding Gaussian and was always chosen such that the Gaussian was white with variance one. 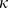 was determined by the 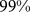-percentile. The only remaining free parameter of the Naka-Rushton distribution is 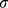 which simultaneously affects both shape and scale. 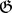 is the regularized-incomplete-gamma function which accounts for the truncation at 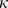. We call the distribution *Naka-Rushton distribution* and denote it with 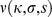.

To derive the distribution on 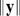 for which the extended divisive normalization transformation 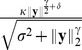 yields a 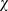-distribution, the steps are exactly the same as for the plain divisive normalization transform above. This yields
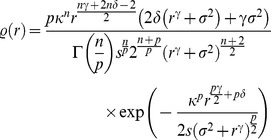
for 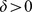. The parameters of the distribution are now 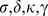 and 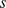.

The parameters for all divisive normalization transforms were estimated via maximum likelihood of the Naka-Rushton distribution on the Euclidean norms 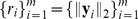 of the filter responses to natural image patches. As before, we did not optimize for 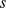 in the extended Naka-Rushton distribution but fixed it such that the corresponding Gaussian was white.

#### Dynamically adapting 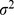


For the model with dynamically adapting 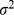, we first model the Euclidean norms 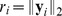 of the filter responses to the patches from the simulated eye movement data with a mixture of 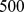 Naka-Rushton distributions
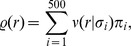
using EM [37]. 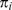 denotes the probability that 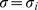. The values of 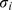 where chosen in 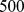 equidistant steps from 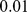 to 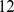.

How much redundancy reduction can be achieved with a dynamically adapting 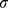, depends on the dynamics according to which it is selected based on the recent history. While there might be many strategies, we chose a parsimonious one based on the mean and the standard deviation of the posterior over 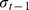. Our heuristic consists of two steps: First the mean and the standard deviation of the posterior 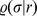 derived from the mixture distribution is approximated with piecewise linear functions 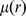 and 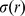, then we sample 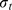 used to transform 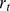 from a 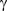-distribution with mean and standard deviation 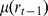 and 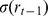. This strategy emphasizes that the first two moments of the posterior are the important features for obtaining a good 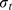.

In more detail, we evaluated the posterior
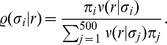
of the mixture distribution at 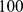 equidistant locations between 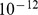 and 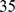, computed the posterior mean and standard deviation at those locations, rescaled the standard deviation by 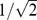, and fitted the piecewise linear functions on the intervals 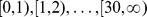 to each set of values. In the first interval, the linear function was constraint to start at zero. From these two functions 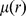 and 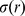, we computed two functions for the scale 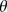 and the shape 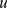 of a 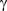-distribution
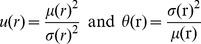
via moment matching. We obtained the value 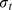 for transforming a value 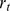 with a Naka-Rushton function by sampling 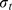 from a 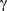-distribution with shape and scale determined by 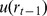 and 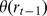.

#### Computation of percentiles for Figure 5

For the dynamically adapting 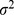 in Figure 5, we sampled from
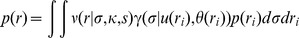
and computed the percentiles based on the sampled dataset. For the sampling procedure, we drew 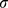 from the 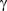-distribution 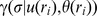 with shape and scale computed from 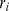 and then sampled 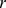 from the Naka-Rushton distribution 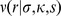 with that 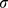. We repeated that for all 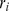 from a test set of simulated eye movement radii. This procedure was carried out for all pairs of training and test sets, and the distributions fitted to them.

For the static case, we sampled data from single Naka-Rushton distributions for different values of 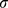 and computed the percentiles from the samples.

#### History-independent choice of 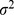


In the following, let 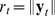 and 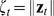 be the unnormalized and normalized responses at time 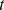, respectively, and 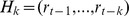 be the recent history of responses. The underlying generative structure of the model for temporally correlated data is the following: given a fixed history 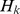, 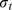 and 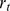 are sampled from 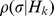 and 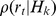. Then, 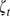 is generated from 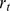 and 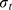 through divisive normalization.

For strong redundancy reduction, 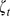 should follow a truncated 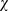-distribution, which means that for given history 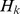 and 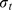, the unnormalized response energy 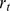 must have a Naka-Rushton distribution
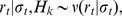
because normalizing this response via 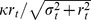 yields a truncated 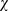-distribution. Averaged over all histories 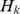 and half-saturation constants 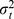 the distribution of 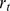 is a mixture of Naka-Rushton distributions
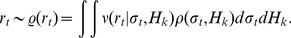
(8)If 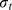 depends deterministically on 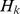 we obtain equation (5).

If 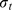 could be chosen independently of the preceding history the distribution of 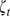 would be given by
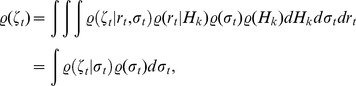
where 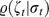 is the marginal distribution of 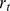 transformed with divisive normalization and a specific value of 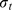. Since redundancy reduction requires 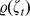 to be truncated 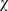-distributed, 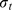 can be chosen independently only if the truncated 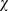-distribution can be modelled as mixture of the different 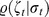. Since we assume stationarity, we can drop the index 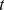 in the equation.

### Multi-information estimation

We use the *multi-information* to quantify the statistical dependencies between the filter responses 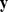 [38]. The multi-information is the 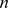-dimensional generalization of the *mutual-information*. It is defined as the Kullback-Leibler divergence between the joint distribution and the product of its marginals or, equivalently, the difference between the sum of the marginal entropies and the joint entropy
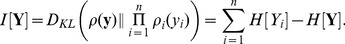
(9)The multi-information is zero if and only if the different dimensions of the random vector 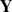 are independent. Since the joint entropy 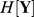 is hard to estimate we employ a semi-parametric estimate of the multi-information that is conservative in the sense that it is downward biased.

For the marginal entropies 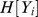, we use a jackknifed estimator for the discrete entropy on the binned values [39]. We chose the bin size with the heuristic proposed by Scott [40]. We obtain an estimate for the differential entropy by correcting with the logarithm of the bin width (see e.g. [7]).

In order to estimate the joint entropy, we use the average log-loss to get an upper bound
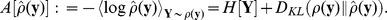
Since the average log-loss overestimates the true entropy, replacing the joint entropy by 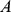 in equation (1) underestimates the multi-information. Therefore, we sometimes get estimates smaller than zero. Since the multi-information is always positive, we set the value to zero in that case. For computing errorbars on the multi-information estimations, we use the negative values but a mean zero in such cases, which effectively increases the standard deviation of the error.

Since we want commit ourselves as little as possible to a particular model, we estimate 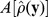 by making the assumption that 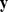 is 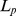-spherically symmetric distributed but estimating everything else with non-parametric estimators. If 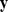 is 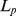-spherically symmetric distributed, the radial component is independent from the directional component [32] and we can write
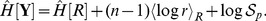
(10)The entropy 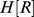 of the radial component is again estimated via a histogram estimator. The term 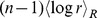 is approximated by the empirical mean.

Putting all the equations together yields our estimator for the multi-information under the assumption of 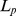-spherically symmetric distributed 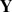


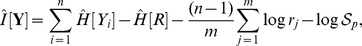
where 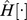 are the univariate entropies estimated via binning.

Since the optimal value of 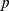 for filter responses 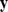 to natural image patches is approximately 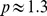 we use that value to estimate the multi-information of 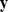.

When estimating the multi-information of the responses 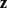 of either divisive normalization or radial factorization, we use the fact that
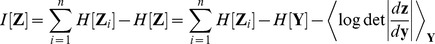
where 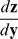 is the Jacobian of the normalization transformation. The mean is estimated by averaging over data points. The determinants of radial factorization, divisive normalization, and extended divisive normalization are given by
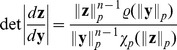


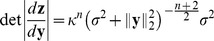


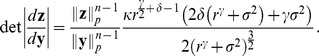
All multi-information values were computed on test data.

For the dynamically adapting model, the 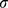 for each data point 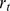 is sampled from a 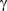-distribution whose parameters are determined from the previous value 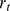 and the posterior over 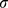 obtained from the mixture of Naka-Rushton distributions. Since 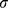 changes from step to step it becomes part of the representation and should be included when computing the multi-information (i.e. the redundancy) between the outputs 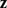. Therefore, the redundancy for the dynamically adapting model is measured by 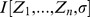. For its computation, we use that 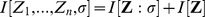, where 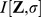 is the mutual information between 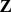 and 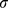. In the following, we write 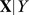 if 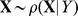. Under the assumption that both 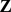 and 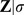 are spherically symmetric distributed, we can decompose respective random variables into the uniform (on the sphere) and the radial part: 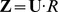 and 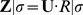. This yields
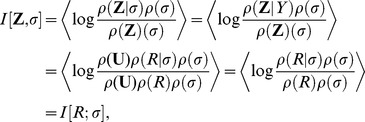
which means that we can restrict ourselves to the mutual information between the two univariate signals 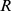 and 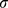, which we estimate from a two-dimensional histogram with 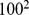 bins.
